# Neuroprotective Effects of Chronic Resveratrol Treatment and Exercise Training in the 3xTg-AD Mouse Model of Alzheimer’s Disease

**DOI:** 10.3390/ijms21197337

**Published:** 2020-10-04

**Authors:** Tom L. Broderick, Suhail Rasool, Rongzi Li, Yuxian Zhang, Miranda Anderson, Layla Al-Nakkash, Jeffrey H. Plochocki, Thangiah Geetha, Jeganathan Ramesh Babu

**Affiliations:** 1 Department of Physiology, College of Graduate Studies, Midwestern University, Glendale, AZ 85308, USA; lalnak@midwestern.edu; 2 Laboratory of Diabetes and Exercise Metabolism, College of Graduate Studies, Midwestern University, Glendale, AZ 85308, USA; manderson92@midwestern.edu; 3 Department of Nutrition, Dietetics, and Hospitality Management, Auburn University, Auburn, AL 36849, USA; suhailrasool@hotmail.com (S.R.); rzl0050@auburn.edu (R.L.); yzz0088@auburn.edu (Y.Z.); thangge@auburn.edu (T.G.); 4 Department of Medical Education, University of Central Florida, College of Medicine, 6850 Lake Nona Blvd, Orlando, FL 32827, USA; Jeffrey.Plochocki@ucf.edu

**Keywords:** Alzheimer’s disease, exercise, resveratrol, brain, neuroinflammation, amyloid-beta, apoptosis, neurotrophin, autophagy

## Abstract

To date, there is no cure or effective treatment for Alzheimer’s disease (AD), a chronic neurodegenerative condition that affects memory, language, and behavior. AD is characterized by neuroinflammation, accumulation of brain amyloid-beta (Aβ) oligomers and neurofibrillary tangles, increased neuronal apoptosis, and loss of synaptic function. Promoting regular exercise and a diet containing polyphenols are effective non-pharmacological approaches that prevent the progression of neurodegenerative diseases. In this study, we measured various conformational toxic species of Aβ and markers of inflammation, apoptosis, endolysosomal degradation, and neuroprotection after 5 months of exercise training (ET), resveratrol (Resv) treatment, or combination treatment in the 3xTg-AD mouse model of AD. Our main results indicate that Resv decreased neuroinflammation and accumulation of Aβ oligomers, increased levels of neurotrophins, synaptic markers, silent information regulator, and decreased markers of apoptosis, autophagy, endolysosomal degradation and ubiquitination in the brains of 3xTg-AD mice. ET improved some markers related to neuroprotection, but when combined with Resv treatment, the benefits achieved were as effective as Resv treatment alone. Our results show that the neuroprotective effects of Resv, ET or Resv and ET are associated with reduced toxicity of Aβ oligomers, suppression of neuronal autophagy, decreased apoptosis, and upregulation of key growth-related proteins.

## 1. Introduction

Alzheimer’s disease (AD) is the most prevalent form of dementia and patients with this neurodegenerative disease exhibit cognitive impairment, memory loss, and behavioral manifestations [1]. According to recent statistics by the World Alzheimer’s Report, nearly 47 million people worldwide are living with dementia and the number of new cases is expected to exceed 130 million by 2050 [2]. AD affects one in seven Americans aged 65 years and the prevalence of this disease will undoubtedly increase with the prolonged life expectancy.

Although the causes of this chronic disease are not fully understood, abnormal accumulation of Aβ into plaques and hyperphosphorylated tau, leading to neurofibrillary tangle (NFT) formation have been identified as the two major hallmarks of AD. The cognitive decline, memory loss, and dementia in AD are also associated with neuroinflammation, oxidative stress, mitochondrial dysfunction, apoptosis, and misfolded proteins [3,4,5]. Additionally, there is currently a substantial number of investigations aimed at understanding the role of folding of aggregated Aβ oligomers, which form prior to the development of plaques [6,7,8]. It has been well-established that aggregated Aβ, particularly in oligomer conformation, contributes to mislocalization and hyperphosphorylation of tau [9,10,11,12,13]. Recently, it has been observed that intraneuronal Aβ accumulates in early events of AD, which can be detected with M78 antibodies but not by regular Aβ antibodies, including 4G8 and 6E10 [14].

Despite the medical and economic significance of AD with a growing aging population, current drug treatment remains inadequate. Exercise is a safe and effective non-pharmacological approach known to delay the progression of neurodegenerative diseases. Exercise training (ET) performed on a regular basis improves the quality of life and reduces the incidence of cognitive defects and the progression of AD [15,16,17]. In the 3xTg mouse model of AD, ET affords neuroprotection by reducing Aβ content [18,19], inflammation and apoptosis in the hippocampus [20,21,22,23] and by improving mitochondrial function and neurogenesis [21]. Similarly, the consumption of natural products containing polyphenols has gained interest as a non-pharmacological approach for the treatment and prevention of AD. Resveratrol (3,5,4-trihydroxy-trans-stilbene, Resv) is a polyphenolic phytoalexin and the main ingredient found in wine, grape seeds, and nuts [24]. Evidence indicates that Resv attenuates learning impairment and delays the onset of neurodegeneration in transgenic murine models of AD [25,26]. In addition, extracts of polyphenolic grape seed attenuate cognitive deficits in Tg256 mice and reduce Aβ oligomer content in the brain [27]. A significant reduction in the number of activated microglia and decreased inflammation in APP/PS1 mice following Resv treatment has been reported [28]. Resv also selectively remodels soluble oligomers and other structures of Aβ into alternative species that are non-toxic [29] and is known to induce the expression of brain-derived neurotrophic factor (BDNF), indicating a beneficial effect on neurotrophin synthesis [30]. In pregnant rats, Resv differentially activates promoters of the BDNF gene [31] and increases the expression of BDNF in the primary culture of neurons and glial cells [32], suggesting a potential role in synaptic plasticity and memory formation [33]. Taken together, the results of these studies clearly highlight the beneficial effects of ET and Resv and their potential use in the treatment of AD.

The effects of Resv treatment in combination with chronic ET, both of which can afford neuroprotection have not been well characterized in the 3xTg mouse model of AD. Recent reports have demonstrated that combined Resv and ET treatment have the advantage of improving both cardiovascular function and fracture resistance in the 3xTg mouse [34,35], suggesting that benefits of such treatment may also occur in other tissues, including the brains of these mice. Therefore, considering the benefits of both ET and the consumption of Resv in the prevention of neurodegenerative diseases, the purpose of this study was to examine the role of ET and dietary supplementation with Resv on different conformational toxic species of Aβ, Aβ-induced apoptosis, inflammation, neuroprotection, and regulation of the endolysosomal pathway in the 3xTg mouse. Since previous studies have indicated that ET alone is protective against neurodegeneration [18,19,20,21,22,23], we aimed to determine whether the combined treatment of ET with Resv supplementation would provide added protection against the development of AD-induced pathology.

## 2. Results

Mice in the ET groups (3xTg-AD and 3xTg-AD + Resv groups) ran without reluctance and completed the exercise protocol. In addition, no signs of poor health or distress were observed in mice treated with Resv. This was confirmed by the observation that there were no differences in food intake expressed in g/kg/day (T, 0.114 ± 0.001; 3xTg-AD, 0.126 ± 0.008; 3xTg-AD + ET, 0.120 ± 0.009; 3xTg-AD + Resv, 0.117 ± 0.007; 3xTg-AD + Resv + ET, 0.136 ± 0.007; F = 1.8738, *p* = 0.1544) and body weight between groups recorded at the end of the treatment protocol (range 35 ± 2 to 39 ± 2 g, NS) [34]. Based on the composition of the diet, Resv intake was estimated at 0.481 g/kg/24-h and 0.557 g/kg/24-h in 3xTg-AD mice + Resv and 3xTg-AD + Resv + ET mice, respectively.

**Figure 1:**
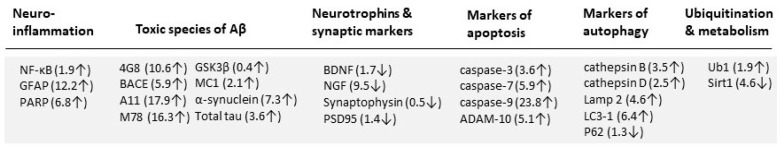
Relative changes in the expression of proteins of interest in brain tissue of 3xTg-AD mice. Changes shown as increased (↑) or decreased (↓) in protein expression in brain of 7-month-old 3xTg-AD mice compared to brain from age-matched WT mice. Numbers in parentheses represent the fold change compared to WT mice. Based on the Western blot data, proteins relating to neuroinflammation, toxic species of Aβ, apoptosis, autophagy and ubiquitination were increased whereas protein levels of neurotrophins, synaptic markers and SIRT1 were reduced in the 3xTg-AD mouse brain compared to age-matched WT mice.

Figure 1 illustrates the protein expression profile in brain tissue of 7-month-old control 3xTg-AD mice compared to brain tissue obtained from age-matched WT controls. Compared to control mice, brain tissue from mice in the 3xTg-AD group exhibited significantly higher levels of proteins linked to neuroinflammation, toxic species of Aβ, apoptosis, autophagy and ubiquitination. However, protein levels of neurotrophins, synaptic markers and SIRT1 were reduced in the brains of 3xTg-AD mice compared to WT mice.

### 2.1. Resv or Resv with ET Reduces Neuroinflammation in 3xTg-AD Mice

**Figure 2:**
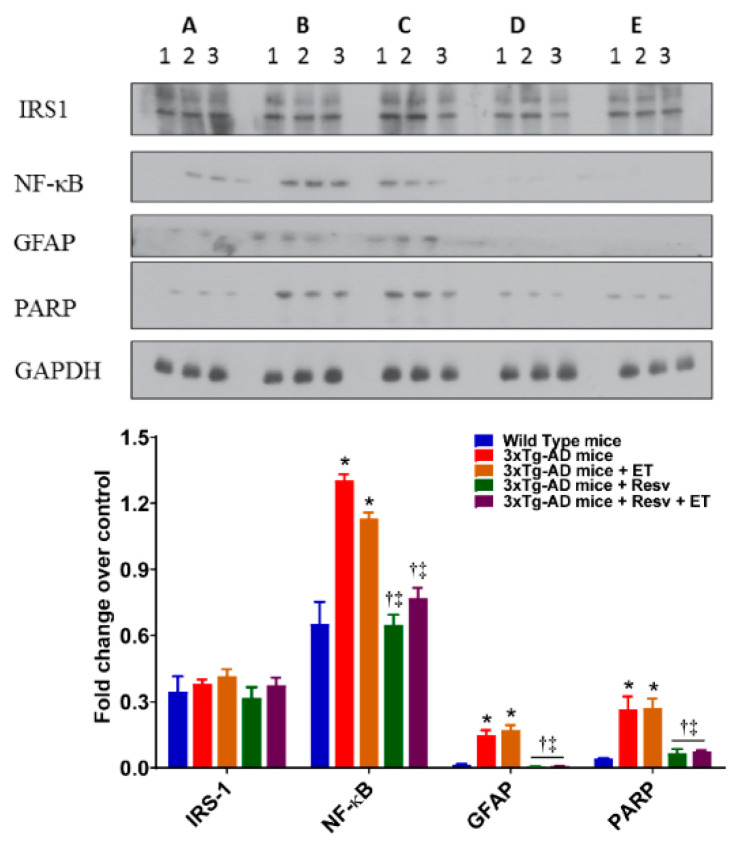
The effects of Resv and exercise training (ET) markers of neuroinflammation. Treating 3xTg-AD mice with Resv or Resv with ET decreases the expression of key inflammatory markers. Corresponding densitometry measurements: A, wild-type (WT) mice; B, 3xTg-AD mice; C, 3xTg-AD mice + ET; D, 3xTg-AD mice + Resv; E, 3xTg-AD mice + Resv + ET. NF-κB, nuclear factor-kappa B; GFAP, glial fibrillary acidic protein; PARP, poly (ADP-ribose) polymerase; IRS-1, insulin-receptor substrate. AD, Alzheimer’s disease; ET, exercise training; Resv, resveratrol. Data are presented as mean ± SEM for 3 mice per group. *, compared to WT mice, *p* < 0.001; ^†^, compared to 3xTg-AD mice, *p* < 0.001; ^‡^, compared to 3xTg-AD mice + ET, *p* < 0.05 for NF-κB.

Inflammation is an important contributor to the pathogenesis of AD. Activation of microglia, astrocytes, and increases in expression levels of inflammatory markers are observed in the brain of AD [36]. As shown in Figure 2, the brains from 3xTg-AD mice exhibited a significant increase in inflammation expressed as NF-κB, GFAP, and PARP. There was no effect of ET on the levels of inflammatory markers. However, treatment with Resv or in combination with ET resulted in a significant decrease in inflammation compared to 3xTg-AD mice.

### 2.2. Resv or Resv with ET Attenuates the Accumulation of Toxic Conformational Species of Aβ

**Figure 3:**
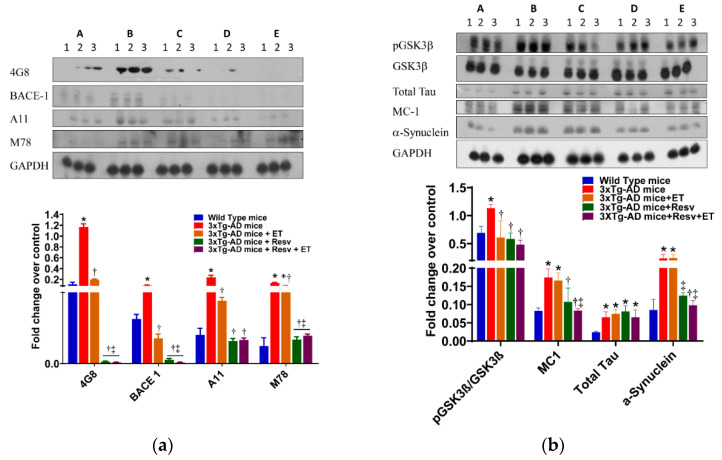
The effects of Resv and ET on conformational toxic species of Aβ. (**a**) Treating 3xTg-AD mice with Resv or Resv with ET reduces extracellular and intracellular Aβ accumulation in brain. Aβ content, oligomers of Aβ and intracellular Aβ detected by A11 and M78 antibody. (**b**) Protein content of pGSK3-β was reduced in 3xTg-AD mice treated with Resv, ET, and Resv with ET. Tau oligomers in brain detected by using the MC1 antibody were also reduced with treatment with Resv or Resv with ET but with no change in total tau. Misfolded protein expression levels using α-synuclein antibody were reduced in 3xTg-AD mice treated with Resv or Resv with ET. Corresponding densitometry measurements: A, WT mice; B, 3xTg-AD mice; C, 3xTg-AD mice + ET; D, 3xTg-AD mice Resv; E, 3xTg-AD mice + Resv + ET. BACE1, beta-secretase enzyme 1; pGSK3β, glycogen synthase kinase beta (*p* = phosphorylated). AD, Alzheimer’s disease; ET, exercise training; Resv, resveratrol. Data are presented as mean ± SEM for 3 mice per group. *, compared to WT mice, *p* < 0.01; ^†^, compared to 3xTg-AD mice, *p* < 0.01; ^‡^, compared to 3xTg-AD mice + ET, *p* < 0.01.

The 3xTg-AD mouse develops age-related progressive neuropathology, including plaques and NFT. At 6 months of age, extracellular Aβ deposits in the frontal cortex are evident. In older mice, as expected, Aβ deposits are increased in this area of the brain [37]. Western blot analysis of conformational antibodies to detect structural aggregates of Aβ was performed in the brain of 3xTg-AD mice. Protein expression levels of Aβ detected by 4G8, BACE 1, A11, and M78 antibodies are illustrated in Figure 3a. As expected, protein expression of 4G8 and BACE 1 was increased in the brain of 3xTg-AD mice. Treatment with Resv or ET was associated with a reduction in 25 kDa molecular weight of Aβ detected by 4G8 and 65 kDa protein detected by BACE-1 antibodies compared to 3xTg-AD mice (Figure 3a). Combination treatment was also effective in reducing 4G8 and BACE 1 protein levels compared to brains from 3xTg-AD mice. M78 stains neuronal nuclei, whereas at a later stage of AD, M78 immunoreactivity localizes with a subset of amyloid plaques that are distinct and shows no immunoreactivity with Aβ or APP antibodies [14]. Protein levels of Aβ oligomers and intracellular Aβ fibrils recognized by A11 and M78 antibody, respectively, were elevated in the brain of 3xTg-AD mice compared to WT mice. ET, Resv, and combination treatment were beneficial in reducing protein levels of different conformation of Aβ recognized by the A11 and M78 antibodies (Figure 3a). Toxic oligomeric species Aβ*56 detected by A11 antibody was significantly reduced by the treatment of Resv. In addition, higher molecular weight fibrils above 200 Kd detected by M78 antibody was significantly reduced with the treatment of Resv.

The brains of untreated 3xTg-AD mice or treated with Resv or in combination with ET showed significantly increased total tau content compared to WT mice. The oligomeric species of tau protein detected by MC1 antibodies in 3xTg-AD mice treated with Resv or in combination with ET were significantly decreased (Figure 3b). Although total tau protein levels were increased in transgenic mice, we did not observe any hyperphosphorylated tau due to their relatively young age, although tau pathology is evident in older 3xTg-AD mice [36,37]. 

Abnormal expression of α-synuclein protein occurs spontaneously in the brain and has been associated with oxidative stress, impaired proteasome function and mitochondrial abnormalities. Oxidative stress can induce aggregation of α-synuclein protein into amyloid-like fibrils [38,39]. Western blot data demonstrated a significant increase in α-synuclein protein expression in brains of 3xTg-AD mice compared to WT mice (Figure 3b). A significant reduction in the accumulation of α-synuclein protein in 3xTg-AD mice detected by α-synuclein antibodies was observed following Resv treatment when compared to 3xTg-AD mice. ET had no effect on protein expression of α-synuclein, but in combination with Resv, protein expression of α-synuclein was significantly decreased. Taken together, our results suggest that Resv can reduce the formation of toxic species of Aβ and tau and prevent the accumulation of misfolded proteins in brains of 3xTg-AD mice. The effects of ET are not as robust as those exhibited with Resv. However, combination treatment appears to be as effective as Resv treatment alone. 

### 2.3. Resv or Resv with ET Increases Protein Expression of Neurotrophins and Synaptic Markers in 3xTg-AD Mice

**Figure 4:**
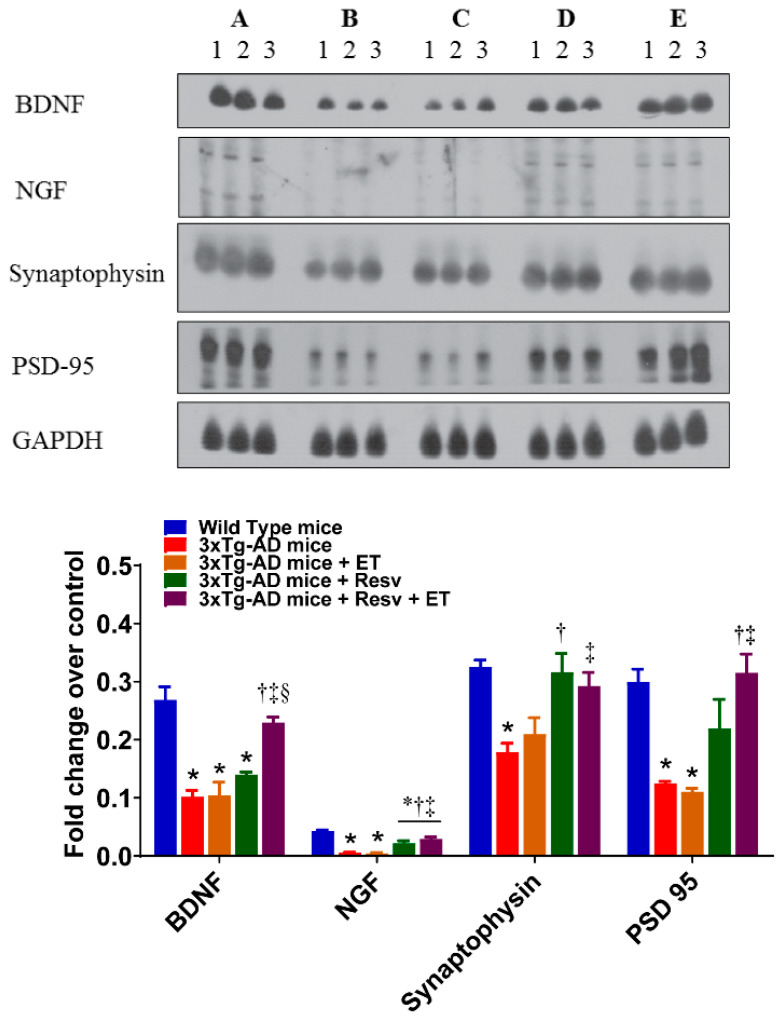
The effects of Resv and ET on the expression of neurotrophins and synaptic markers. Treatment with Resv or Resv with ET increased the expression of neurotrophins and synaptic markers. Representative immunoblots show significant increases in expression of the neurotrophins BDNF, NGF and synaptic markers synaptophysin and PSD-95 in 3xTg-AD mice treated with Resv or Resv with ET. Corresponding densitometry measurements: A, WT mice; B, 3xTg-AD mice; C, 3xTg-AD mice + ET; D, 3xTg-AD mice + Resv; E, 3xTg-AD mice + Resv + ET. BDNF, brain-derived neurotropic factor; NGF, nerve growth factor; PSD95, postsynaptic density 95. AD, Alzheimer’s disease; ET, exercise training; Resv, resveratrol. Data are presented as mean ± SEM for 3 mice per group. *, compared to WT mice, *p* < 0.05; ^†^, compared to 3xTg-AD mice, *p* < 0.05; ^‡^, compared to 3xTg-AD mice + ET, *p* < 0.05; ^§^, compared to 3xTg-AD mice + Resv.

In addition to the presence of plaque and NFT pathology, low levels of neurotrophins and synaptic markers contribute to the pathogenesis of AD. The effects of treatment on the protein expression of neuronal and synaptic markers are illustrated in Figure 4. Our results show that protein levels of BDNF, NGF, synaptophysin, and PSD-95 detected by respective antibodies were significantly reduced in the brain of 3xTg-AD mice compared to WT mice. ET had no effect on the protein expression of neuronal and synaptic markers. However, treatment with Resv increased protein expression of NGF and synaptophysin. Protein levels of BDNF, NGF, synaptophysin and PSD-95 were significantly increased following combined ET and Resv treatment compared to 3xTg-AD mice. Therefore, our results indicate that either Resv treatment alone or with ET exerts beneficial effects on the expression of neurotrophins and synaptic markers.

### 2.4. Resv or Resv with ET Reduces Apoptosis and Decreases Protein Expression of Autophagy and Accumulation of Ubiquitinated Proteins

**Figure 5:**
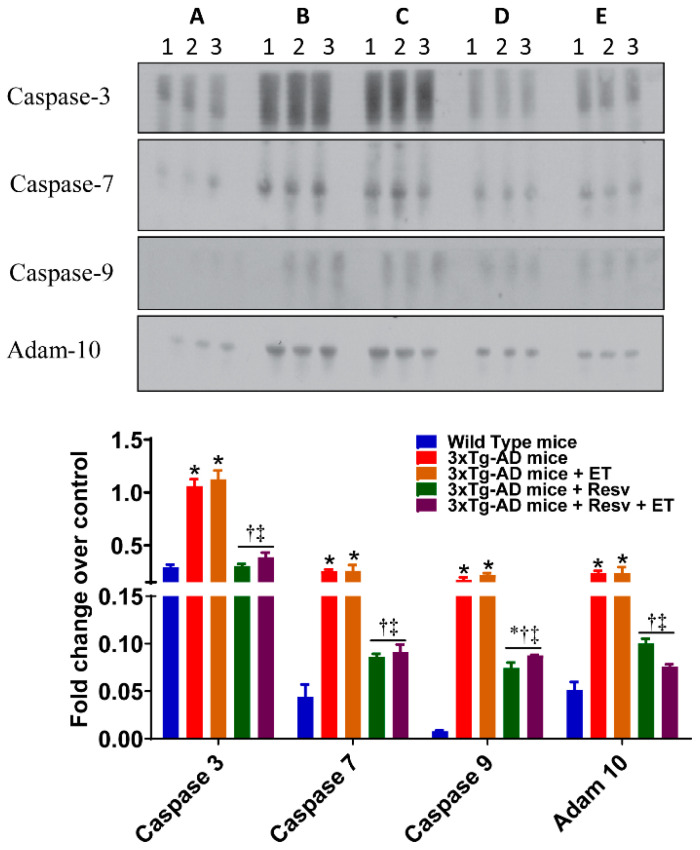
The effects of Resv and ET on the expression of markers of apoptosis. Treatment with Resv or Resv with ET decreased the expression of markers of apoptosis. Representative immunoblots show significant decreases in the expression of caspase 3, 7, 9, and Adam 10. Corresponding densitometry measurements: A, WT mice; B, 3xTg-AD mice; C, 3xTg-AD mice + ET; D, 3xTg-AD mice + Resv; E, 3xTg-AD mice + Resv + ET. Adam 10, disintegrin and metallopeptidase domain-containing protein 10. AD, Alzheimer’s disease; ET, exercise training; Resv, resveratrol. Data are presented as mean ± SEM for 3 mice per group. *, compared to WT mice, *p* < 0.05; ^†^, compared to 3xTg-AD mice, *p* < 0.05; ^‡^, compared to 3xTg-AD mice + ET, *p* < 0.05.

Aβ and hyperphosphorylated tau lead to apoptosis and autophagy, which ultimately causes cognitive dysfunction in AD. The effects of treatment on the expression of markers of apoptosis are shown in Figure 5. The protein content of caspase-3, caspase-7, caspase-9, and adam-10 was increased in the brain from 3xTg-AD mice compared to WT mice. Treatment with Resv resulted in a significant decrease in apoptosis when compared to 3xTg-AD mice (detected by antibodies specific for activated caspases), while ET had no effect. However, combined treatment was just as effective as Resv on protein expression of these markers of apoptosis. 

**Figure 6:**
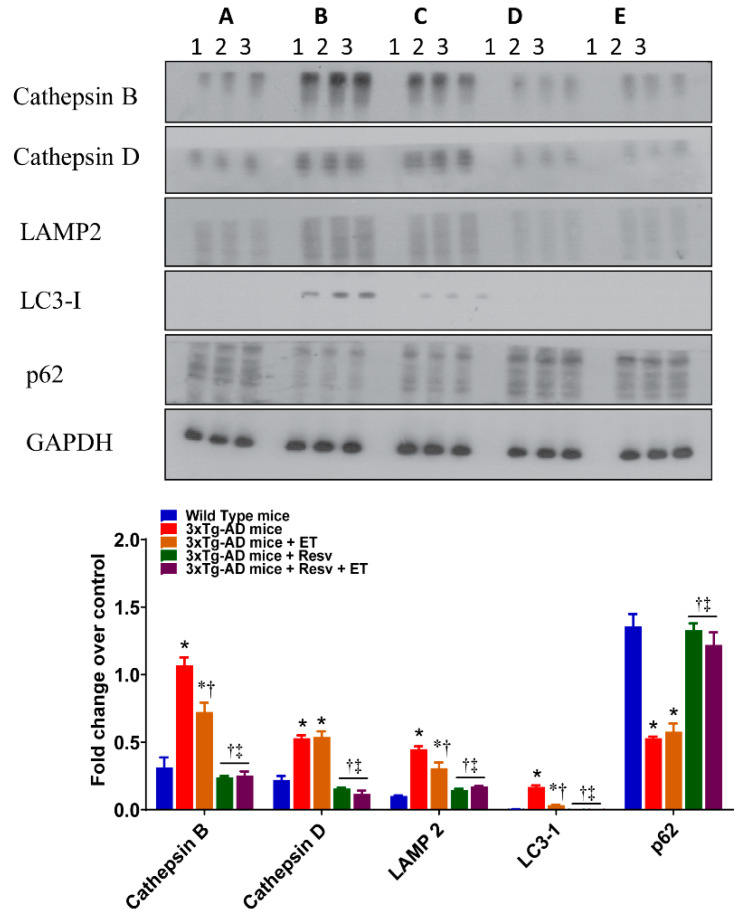
The effects of Resv and ET on the expression of markers of autophagy. Treatment with Resv or Resv with ET decreased protein expression of key autophagy markers. Autophagy was expressed using LC3-1 and Cathepsin B, Cathepsin D, and LAMP2 were measured as endolysosomal markers. Representative immunoblots show significant reductions in the expression of all markers with ET, Resv, and Resv with ET. The significant decrease in the expression p62 in brains of 3xTg-AD mice was prevented with Resv or Resv with ET. Corresponding densitometry measurements: A, WT mice; B, 3xTg-AD mice; C, 3xTg-AD mice+ET; D, 3xTg-AD mice + Resv; E, 3xTg-AD mice + Resv + ET. LAMP2, lysosomal-associated membrane protein; LC3-1, microtubule-associated protein 1 light chain 3 beta; p62, sequestosome 1/ubiquitin-binding protein. AD, Alzheimer’s disease; ET, exercise training; Resv, resveratrol. Data are presented as mean ± SEM for 3 mice per group. *, compared to WT mice, *p* < 0.05; ^†^, compared to 3xTg-AD mice, *p* < 0.05; ^‡^, compared to 3xTg-AD mice + ET, *p* < 0.05.

Protein expression levels of autophagy detected by antibodies for LC3-1 and antibodies for the endolysosomal proteins cathepsin B, cathepsin D, and LAMP2 are illustrated in Figure 6. Brain from 3xTg-AD mice expressed significantly higher protein expression levels of cathepsin B and D, LAMP2, and LC3-1 compared to WT mice. ET significantly reduced protein levels of cathepsin B, LAMP2, and LC3-1 when compared to the brains of 3xTg-AD mice. Treatment with Resv and combination treatment were both associated with a greater reduction in the expression as detected by these antibodies. 

Protein turnover by autophagy and the ubiquitin–proteasome system is mediated, in part, by p62 [40,41]. The role of this multifunctional protein is supported by the observation that p62 knockout mice exhibit an accumulation of NFT associated with defects in synaptic function [42]. The effects of treatment on p62 protein expression are shown in Figure 6. Compared to WT mice, the brains of 3xTg-AD mice demonstrated a significant reduction in p62. This reduction was reversed in mice treated with Resv and with combined treatment.

**Figure 7:**
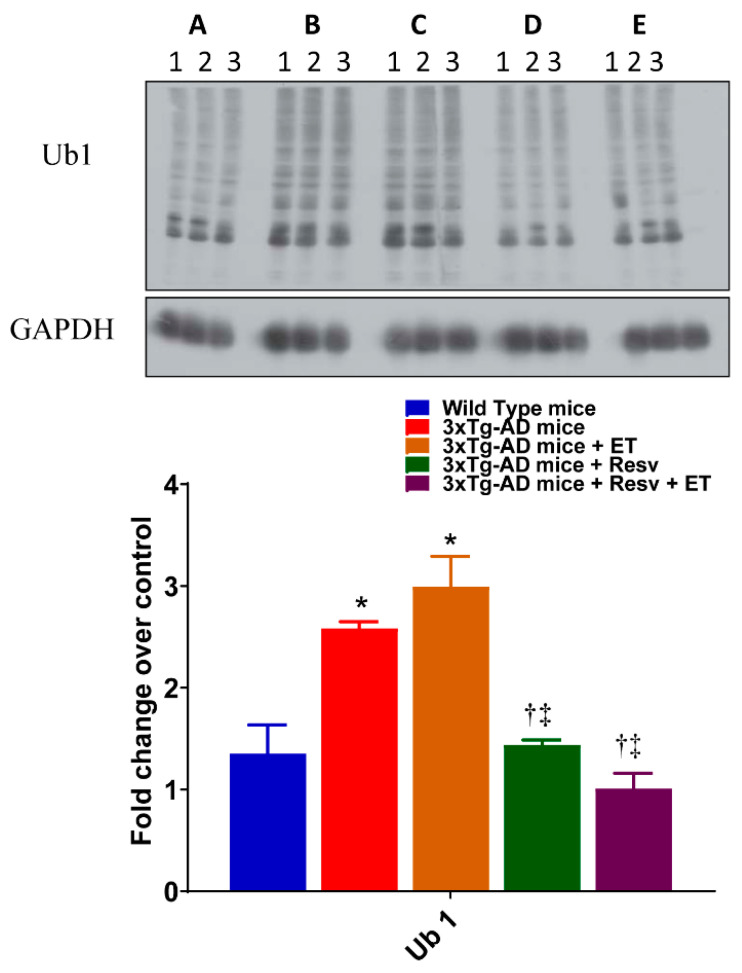
The effects of Resv and ET on protein ubiquitination. Treatment with Resv or Resv with ET decreased protein ubiquitination, expressed as Ub1. Corresponding densitometry measurements: A, WT mice; B, 3xTg-AD mice; C, 3xTg-AD mice + ET; D, 3xTg-AD mice + Resv; E, 3xTg-AD mice + Resv + ET. AD, Alzheimer’s disease; ET, exercise training; Resv, resveratrol. Data presented as mean ± SD for 3 mice per group. *, compared to WT mice, *p* < 0.05; ^†^, compared to 3xTg-AD mice, *p* < 0.05; ^‡^, compared to 3xTg-AD mice + ET, *p* < 0.05.

The effects of treatment on protein ubiquitination were examined. As shown in Figure 7, a significant increase in the protein expression of ubiquitinated proteins using Ub1 was observed in brains of 3xTg-AD mice compared to WT mice. A similar increase in protein expression of ubiquitinated protein Ub1 was observed in brains of 3xTg-AD mice after ET compared to WT mice. However, the level of ubiquitinated proteins was normalized with Resv and with combination treatment. Taken together, our results indicate that treating 3xTg-AD mice with Resv or in combination with ET may prevent apoptosis and autophagy and reduce the accumulation of misfolded and ubiquitinated proteins.

### 2.5. Resv or Resv with ET Increases the Expression Level of SIRT1

**Figure 8:**
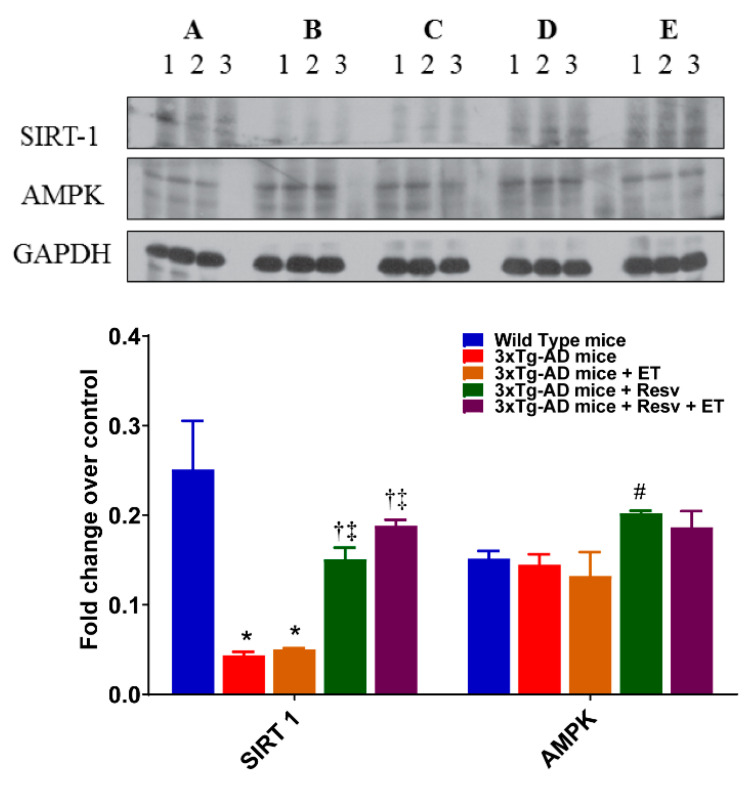
The effects of Resv and ET on protein expression of SIRT1 and AMPK. Representative immunoblots show a significant decrease in the expression level of SIRT1 in 3xTg-AD mice. Treatment with Resv or Resv with ET increased the expression SIRT1. Expression levels of AMPK were not significantly different between groups. Corresponding densitometry measurements: A, WT mice; B, 3xTg-AD mice; C, 3xTg-AD mice + ET; D, 3xTg-AD mice + Resv; E, 3xTg-AD mice + Resv + ET. SIRT1, silent information regulator 1; AMPK, AMP-activated protein kinase. AD, Alzheimer’s disease; ET, exercise training; Resv, resveratrol. Data presented as mean ± SEM for 3 mice per group. *, compared to WT mice, *p* < 0.05; ^†^, compared to 3xTg-AD mice, *p* < 0.05; ^‡^, compared to 3xTg-AD mice + ET, *p* < 0.05; ^#^, *p* = 0.0578 compared to WT mice.

The effects of Resv and ET on SIRT1 protein expression are illustrated in Figure 8. Western blot analysis revealed decreased protein expression of SIRT1 in 3xTg-AD mice compared to WT mice. The expression of SIRT1 also remained low in the brain of mice after ET. However, following treatment with Resv or in combination with ET, SIRT1 protein expression was significantly increased compared to 3xTg-AD mice. No differences in the expression of AMPK between groups were observed.

**Figure 9:**
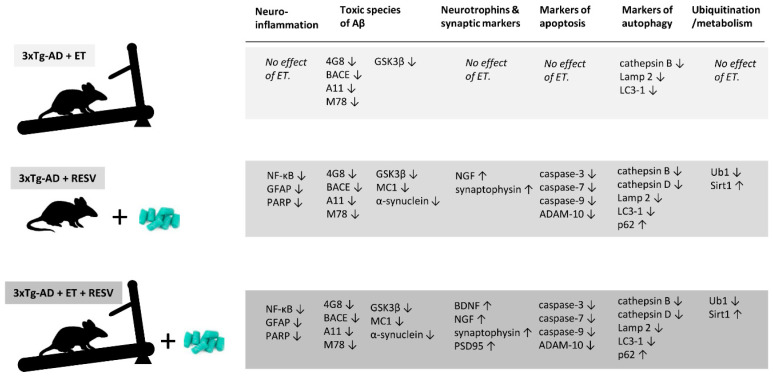
Summary of the effects of Resv and ET on proteins of interest. Changes are expressed as increased (↑) or decreased (↓) based on the Western blot data compared to brain tissue obtained from 3xTg-AD mice serving as controls (Figure 1). The greatest protection was afforded by treatment with Resv alone or by Resv combined with ET.

A summary of the effects of treatment on the expression of proteins or markers of concern is illustrated in Figure 9. Changes in protein expression are compared to brains of mice from the control 3xTg-AD group. Of the 27 proteins examined, 8 were improved with ET, 23 with Resv, and 25 proteins of interest were beneficially affected by combined ET and Resv treatment. ET did not improve the expression of neuroinflammatory, synaptic, and apoptotic markers. However, some markers of autophagy were improved and toxic species of Aβ were reduced with ET. Resv treatment or in combination with ET afforded the greatest benefits on the expression of proteins.

## 3. Discussion

It is well-established that exercise and healthful eating are protective against the development and progression of various age-related and neurodegenerative diseases. Performing aerobic exercise on a regular basis and the consumption of a diet rich in polyphenols are not only associated with fewer side effects and better adherence but also improve the quality of life in patients with progressive neurodegenerative diseases. These non-pharmacological strategies are known to preserve the blood–brain barrier (BBB), improve CNS immunity, reduce hippocampal atrophy, and improve blood flow and cognitive function, and functional ability [43,44,45]. In this study, we examined the effects of chronic Resv treatment and ET in the form of treadmill running on common markers of AD-induced pathology in the brain. We used the 3xTg-AD mouse model because of its close representation of the human condition. To reflect the early stages in the development of AD in the absence of significant tau pathology [37], treatment was initiated in 2-month-old mice and continued for a period of 5 months. Our analysis of brain tissue from 3xTg-AD mice revealed, as expected, severe pathologic disturbances that are not only consistent with earlier studies using this model, but also reminiscent of the human condition of AD. Brains from 3xTg-Ad mice displayed neuroinflammation, accumulation of toxic species of Aβ, increased apoptosis and autophagy, and decreased expression of synaptic markers. In terms of the effects of treatment, our data show that: (1) ET decreased the accumulation of Aβ oligomers and markers of autophagy, (2) Resv reduced markers related to inflammation, toxic species of Aβ, apoptosis, autophagy, ubiquitination and endolysosomal degradation, (3) Resv increased the expression of neurotrophins and SIRT1, and (4) combined ET and Resv was as effective and beneficial as Resv treatment alone except for an increase in synaptophysin and PSD95 synthesis. Our results support the relationship between regular exercise and polyphenol consumption and neuroprotection in the 3xTg-Ad mice [18].

The deficit in cognitive function and behavioral changes in patients with AD is associated with an inflammatory response involving microglia, astrocytes, and macrophages [46]. Aggregation of Aβ leads to the activation of microglia and causes the release of proinflammatory mediators including cytokines, free radicals, which in turn increase Aβ production [28,47,48]. Previous studies suggested that the formation of Aβ annular protofibrils (APFs) in astrocytes is linked to the pathogenesis of AD and prevention of these APF formations could be a relevant target for the prevention of Aβ toxicity in AD [49]. Resv has been shown to exert anti-inflammatory properties by reducing the production of proinflammatory markers and suppressing the activation of microglia and astrocytes [50]. Our findings support and extend previous studies on the benefits afforded by Resv against the progression of AD. However, ET alone was not accompanied by a reduction in the expression of neuroinflammatory markers in brain tissue. This is in contrast to previous studies that have demonstrated protection with ET following forced treadmill exercise [21], voluntary wheel running exercise [22] and loaded resistance training [20] in the 3xTg-AD mouse. Decreases in protein expression of TNF-α, IL-6, and GFAP in the hippocampus, cortex, and hypothalamus were seen under these training conditions, resulting in an improvement in cognitive function [20,21]. In addition, treadmill running suppressed the accumulation of neuroinflammatory markers in the hippocampus of 3xTg-AD mice fed an obesogenic diet consisting of high fat [23], which is associated with increased risk of AD and known to hasten the development of AD-related neuropathology [51,52].

The reasons for these discrepancies in protection against the increase in neuroinflammatory markers with treadmill running are not known but may relate to differences in the age of mice at the start of the exercise program as well as the intensity of exercise. Where benefits were reported with treadmill exercise, protection against neuroinflammation was achieved at a lower exercise intensity [21], which likely produced a lower stress response. Higher exercise intensity elicits specific negative adaptations, including adrenal hypertrophy, increased corticosterone secretion, and suppressed antigen-specific IgM production [53]. An increased stress response is also known to contribute to the development of age-related neurodegenerative diseases and AD [54,55]. Despite the lack of effect of ET on inflammatory markers in our study, most reports support the premise that ET, regardless of the paradigm, exerts an important role in suppressing neuroinflammation in 3xTg-AD mice. 

In addition to the accumulation of Aβ into plaques and hyperphosphorylated tau leading to NFT, the formation of aggregated Aβ prior to the formation of plaque has been recognized as major hallmarks of AD [6,7,8]. Similarly, accumulation of tau oligomers in the synaptic space leads to synaptic dysfunction, amnesia, and neurodegeneration [56]. Emerging evidence also suggests the involvement of disrupted synaptic vesicle cycle function, which is a site of both Aβ production and toxicity, in the pathology of AD [57]. In addition, the ectopic release of glutamate near axonal swellings surrounding amyloid plaques can lead to increased glutamatergic activity [58]. Aggregated Aβ oligomer conformation and its contribution to mislocalization and hyperphosphorylation of tau have been well established [9,10,11,12,13]. Recently, it has been observed that interneuronal Aβ accumulates in the early development of AD and is detected by M78Ab, but not with regular Aβ antibodies such as 4G8 and 6E10 [14]. Studies suggest that Resv has a potential role in the treatment of hippocampal neurons against Aβ-induced toxicity and neurotoxicity induced by oxidative stress [59,60]. In rats with AD, this effect appears to be mediated by the ability of Resv to cross the BBB [61,62] and increase the expression of antioxidant mechanisms [63,64,65] and reduce the production of inflammatory markers in the brain. Resv may have beneficial effects by inhibiting the extension and destabilizing the polymerization of Aβ peptides and inhibiting Aβ cytotoxicity [66]. Soluble oligomers are toxic and distinct from monomers or fibril aggregates identified in AD brains [67]. During the early progression of AD pathogenesis, Aβ, APP or APP-CTFs (carboxy terminal of amyloid precursor protein) accumulate and aggregate intracellularly (usually in the perinuclear compartment) into a conformation that is recognized by M78Ab and colocalizes with Aβ and APP-CTF [14]. At intermediate stages of plaque pathology, M78 stains neuronal nuclei, whereas at a later stage M78 immunoreactivity localizes with a subset of amyloid plaques that are distinct and show no or less immunoreactivity with Aβ or APP antibodies [14]. This suggests that neuritic plaques arise from degenerative neurons with intracellular immunoreactivity. Our data show a significant reduction in M78, A11, and BACE1 expression in brains of 3xTg-AD mice following Resv treatment. This is consistent with the decreases in expression levels of tau and phosphorylated tau recently reported with Resv [68]. Further, we found that ET also alleviated the AD-induced increases in M78, A11, and BACE1 expression. Although we are the first to document the beneficial effects of ET on toxic species of Aβ detected by these antibodies, other studies nonetheless support the role of exercise in alleviating Aβ pathology. Five weeks of treadmill running prevented the increase in Aβ42/40 ratio from occurring in the cerebral cortex of 7-month-old 3xTg-AD mice [69]. Voluntary wheel running also ameliorated Aβ levels in brains of 3xTg-AD mice although it appears that this effect is dependent on the age of mice at the onset of exercise training [18]. Volitional running initiated in 4-month-old 3xTg-AD mice for a period of 4 weeks was associated with a reduction in soluble Aβ40 levels in the hippocampus whereas no reduction was observed with training initiated in 7-month-old mice after 6 months of training. Recent studies have also supported these beneficial effects of treadmill or wheel running initiated in young mice on AD-related pathology, including decreases in Aβ 1–40 and 1–42 in the hippocampus and cerebral cortex after 20 weeks of treadmill running initiated in 4-month-old 3xTg-AD mice fed a high-fat diet [23] and after 12 weeks of treadmill running in 3 month-old 3xTg-AD mice [21]. In addition to the benefits afforded by aerobic exercise training, resistance training attenuates the amyloid burden in the 3xTg-AD mouse regardless of the age of mice at the onset of exercise [19,20]. It is evident from these studies that exercise, regardless of the form, slows the accumulation of Aβ species. ET combined with Resv affords greater protection than ET alone.

In addition to the role of Aβ, the pathogenesis of AD involves the hyperphosphorylation of tau. Evidence suggests that tau also undergoes differential structural changes to the oligomeric state before the formation of tau fibrils [56,70,71]. Tau oligomers are neurotoxic and have been isolated at early stages and prior to the onset of clinical symptoms in AD patients [56,72,73]. Our results indicate that Resv treatment reduced tau oligomers using MC1 Ab when compared to 3xTg-AD mice, indicating neuroprotection from the accumulation of abnormal proteins. In addition, the effects of ET on Aβ accumulation and tau oligomer expression are in line with early studies indicating a lowering effect in the brains of 3xTg-AD mice. 

The involvement of neurotrophic and synaptic markers in the pathogenesis of AD has been previously described [74]. Studies have shown that plasma levels of BNDF and gene and protein expression of BDNF in the hippocampus and cortex are reduced in AD brains [75,76,77,78,79]. BDNF acts as an important mediator of synaptic plasticity and memory formation [33]. NGF plays a role in aging and age-related neurodegenerative diseases because of the presence of abnormalities in trophic signaling [80,81]. In addition, lower levels of PSD95 were detected in AD because of its role as a major scaffolding protein of the dendritic spines and in trafficking of glutamate receptors, ion channels, and adhesion molecules [82,83,84,85,86]. Synaptic plasticity is a cellular substrate for learning and memory and synaptic loss may contribute to impaired synaptic plasticity in AD [87,88,89]. Levels of BDNF in serum and brain tissue are increased with ET [23,90] preventing loss of synaptic stability and function and cell death. In our study, we found that ET alone had no beneficial effect on the expression of BDNF, synaptophysin, and PSD95 in brains of 3xTg-AD mice. In contrast, other studies have reported the benefits of ET on these proteins with both treadmill running and resistance training [21,23]. Those studies also report increases in the levels of synaptotagmin-1 and synaptobrevin-1 following ET. However, it should be emphasized that the increase in these markers was observed in specific areas of the brain, including the hippocampus and cerebral cortex, while our results were obtained from crude whole brain homogenates. This could explain why no significant changes were detected after 5 months of ET in our study whereas localized increases in the hippocampus were seen just after 12 weeks of training. That is, our current findings do not suggest a lack of effect of ET on the expression of the markers but rather caution the interpretation of results based on regions of the brain analyzed. On the other hand, we found that 3xTg-AD mice treated with Resv alone or combined with ET showed an improvement in the expression of several markers compared to control 3xTg-AD mice (Figure 9). Treatment with Resv improved levels of NGF and synaptophysin while Resv in combination with ET provided additional benefits by also increasing the expression of BDNF and PSD95. The observation that the reduction in protein levels of these neurotrophins was prevented suggests that these interventions offer a robust neuroprotective role. 

The accumulation of Aβ may cause both neuronal and synapse loss from apoptosis and the role of resveratrol in reducing apoptotic markers has been reported [30]. Our data demonstrate that 3xTg-AD mice treated with Resv show significant reductions in the expression levels of apoptotic markers. Further, dysregulation in the neuronal endocytic pathway occurs prior to the accumulation of Aβ and tau is considered a seminal event in the pathogenesis of AD [91]. The endosomal–lysosomal system plays an important role since this system is involved in APP processing, uptake of Aβ and its accumulation. Studies have shown an association between impaired autophagy induction and the increase in autophagy suppressing molecules with AD [92,93,94]. Autophagosome/lysosome and the UPS are the two major proteolytic systems in the brain. Endolysosomes play a critical role in amyloidogenic processing of APP [95,96] and Aβ acts a substrate for autophagy [97]. Our results show that 3xTg-AD mice treated with Resv show a significant reduction in the expression levels of endolysosomal and autophagic markers. Defects in Ub-mediated protein degradation and increased levels of Ub-conjugated proteins may also explain the increased formation of NFT [98]. The role of p62, which contains ubiquitin-binding protein and is involved in autophagy [40,99,100], should not be ruled out as reports have demonstrated that the levels of NFT are increased in the brains of p62 knockout mice [42]. For degradation, p62 binds to NFT, which accumulates after phosphorylation of tau. Sequestration of p62 in NFT limits its cellular function by creating a reduced pool of p62 in the cytosol [101]. Our results show that treating 3xTg-AD mice with Resv leads to a significant increase in p62 protein levels. Resv alone or in the presence of ET significantly reduced the expression levels of ubiquitinated proteins, suggesting improved degradation and disposal of proteins.

## 4. Materials and Methods 

### 4.1. Mouse Model of AD and Treatment Protocols

The animal protocols described in this study were approved by the Midwestern University Institutional Animal Care and Use Committee and adhered to the guidelines in the National Institute of Health’s Guide for the Care and Use of Laboratory Animals (Publ. No. 85–23, 1986). Male triple transgenic mice (3xTg-AD, Jackson Laboratories, Bar Harbor, ME, USA), aged 8 weeks, were used in this study. This mouse model of AD, which harbors three mutant genes (Aβ precursor protein, presenilin-1, tau) was selected because of its similarity to the human condition of familial AD [36,37]. Aged-matched, non-transgenic wild type (B6129SS2/J) mice were used as controls. Male mice were assigned to five groups (*n* = 8/group): (1) wild type control (WT), (2) AD control (3xTg-AD), (3) 3xTg-AD exercise training (3xTg-AD + ET), (4) 3xTg-AD resveratrol-treated) and (5) 3xTg-AD resveratrol-treated and exercise (3xTg-AD + Resv + ET).

Mice were trained using an electrical treadmill (Exer 3/6, Columbus Instruments, Columbus OH, USA) using an incremental training protocol. Mice were first acclimated to daily 10-min exercise sessions at 10m/min for a period of two weeks. After this period of acclimation, training consisted of the following as described earlier [34]: week 1, 20 min at 10 m/min; week 2, 30 min at 12 m/min. At week 3, the duration was increased to 45 min and intensity to 15 m/minute corresponding to ~80% maximal oxygen-carrying capacity based on treadmill belt speed [102]. 

Resv (synthetic, 99% Lallilab Inc. Durham, NC, USA) was incorporated into food pellets (Dyets Inc. Bethlehem, PA, USA, Dyet #102270, modified AIN-93G purified rodent diet with 4 g/kg resveratrol). This diet was selected based on earlier studies showing cardioprotective, anti-inflammatory, and neuroprotective effects without any negative effects [34,103,104]. Mice in the WT, AD-3xTg, and 3xTg-AD + ET groups received the same purified diet without the addition of Resv. Mice were housed two per cage and maintained in a room with a 12:12 h light–dark cycle and given food and water ad libitum. Mice were treated with Resv, ET, or both for a period of 5 months. Following treatment, mice were euthanized by CO2 gas followed by cervical dislocation. Brain tissue was harvested, quickly frozen in liquid nitrogen and stored at −80 °C. 

### 4.2. Western Blot Analysis

Analysis was performed using soluble fractions isolated from brains using a method described previously [105]. Briefly, brain tissue was homogenized in Triton lysis buffer containing the following: 50 mM Tris–HCl (H 7.5), 150 mM NaCl, 10 mM NaF, 0.5% Triton X-100, 1 mM Na3VO4, 1 mM phenylmethylsulfonyl fluoride, 2 μg/mL leupeptin, aprotinin and protease inhibitor cocktail. Homogenates were then centrifuged at 14,000 rpm for 1 hr at 4 °C. The supernatants were collected and the concentration of protein in lysates was measured using standard techniques (Pierce 660-nm protein assay reagent, Thermo Scientific, Rockford, IL, USA). Protein (20 µg) from each fraction was resolved by SDS–PAGE and transferred onto polyvinylidene difluoride membranes. Antibodies against BACE-1, A11, tau, MC-1, PARP, GFAP, BDNF, NGF, synaptophysin, PSD-95, caspase (3, 7, 9), Adam10, and SIRT1 were purchased from Abcam, Cambridge, MA, USA. Antibodies against pGSK3β, α-synuclein, KF-κB, LC3-1, p62, and AMPK were obtained from Cell Signaling Technology, Danvers, MA). Antibodies against IRS-1, LAMP8, and Ub1 were purchased from Santa Cruz Biotechnology, Santa Cruz, CA. GAPDH was used as an internal control (Abcam, Cambridge, MA, USA). All other chemicals were purchased from Sigma-Aldrich (St-Louis, MO, USA). The immunoreactive bands were visualized with an enhanced chemiluminescence reagent. Aβ conformation antibodies A11 and M78 were a generous gift from Dr. Charles Glabe of UC Irvine. Western blot images were quantified using image ISO lite software

### 4.3. Statistical Analysis 

Quantification of Western blot data was performed by using Li Core Image J software and data were analyzed using Graph pad prism. A one-way analysis of variance followed by the Tukey–Kramer post hoc test was used to determine differences in group means. All values are reported as mean ± SEM and significance set at *p* < 0.05.

## 5. Conclusions

In conclusion, we are the first to document the effects of ET, Resv treatment, and combination treatment on markers of inflammation, apoptosis, endolysosomal degradation, and neuroprotection as well as conformational toxic species of Aβ in the brain of the 3xTg mouse model of AD. We report that the 3xTg-AD mouse exhibits many of the aberrations in protein synthesis of these key markers that are consistent with neurodegeneration associated with AD. Our data indicate that ET, intake of a diet rich in Resv, or Resv with ET are beneficial in this model of AD. ET afforded a level of neuroprotection marked by a reduction in the toxic species of Aβ and markers of autophagy. Markers of neuroinflammation, synaptic function, apoptosis, and ubiquitination were not improved by ET alone. However, robust protection against the sequelae in this model of AD was observed with Resv and Resv combined with ET. A reduction in neuroinflammation, apoptosis, amyloid burden, tau phosphorylation, and an increase in the synthesis of key neurotrophins was observed. Our data highlight the importance of non-pharmacological approaches in the form of exercise and diet as a mean to promote brain health and delay age-related diseases.

## Abbreviations

Adam 10	disintegrin and metallopeptidase domain-containing protein 10
AD	Alzheimer’s disease
Aβ	amyloid-beta
Ab	anti-body
AMPK	AMP-activated protein kinase
APP	amyloid precursor protein
APP-CTF	(carboxy terminal of amyloid precursor protein)
BACE1	beta-secretase enzyme 1
BBB	blood–brain barrier
BDNF	brain-derived neurotropic factor
ET	exercise training
GFAP	glial fibrillary acidic protein
GSK3β	glycogen synthase kinase beta
IRS-1	insulin-receptor substrate
NF-κB	nuclear factor-kappa B
NGF	nerve growth factor
NFT	neurofibrillary tangles
PARP	poly (ADP-ribose) polymerase
PSD95	postsynaptic density 95
Resv	resveratrol
SIRT1	silent information regulator 1
UB	ubiquitinated proteins
UPS	ubiquitinated proteasome system

## References

[1] Sperling R.A., Dickerson B.C., Pihlajamaki M., Vannini P., LaViolette P.S., Vitolo O.V., Hedden T., Becker J.A., Rentz D.M., Selkoe D.J. (2010). Functional Alterations in Memory Networks in Early Alzheimer’s Disease. Neuromol. Med..

[2] World Alzheimer Report 2018. https://www.alz.co.uk/research/world-report-2018.

[3] Harris M.E., Hensley K., Butterfield D.A., Leedle R.A., Carney J.M. (1995). Direct evidence of oxidative injury produced by the Alzheimer’s beta-amyloid peptide (1-40) in cultured hippocampal neurons. Exp. Neurol..

[4] Eckert A., Hauptmann S., Scherping I., Rhein V., Müller-Spahn F., Götz J., Müller W.E. (2008). Soluble beta-amyloid leads to mitochondrial defects in amyloid precursor protein and tau transgenic mice. Neurodegener. Dis..

[5] Kolarova M., García-Sierra F., Bartos A., Ricny J., Ripova D. (2012). Structure and pathology of tau protein in Alzheimer disease. Int. J. Alzheimers Dis..

[6] Klein W.L. (2013). Synaptotoxic amyloid-β oligomers: A molecular basis for the cause, diagnosis, and treatment of Alzheimer’s disease?. J. Alzheimers Dis. JAD.

[7] Kayed R., Lasagna-Reeves C.A. (2013). Molecular mechanisms of amyloid oligomers toxicity. J. Alzheimers Dis. JAD.

[8] Hardy J., Selkoe D.J. (2002). The amyloid hypothesis of Alzheimer’s disease: Progress and problems on the road to therapeutics. Science.

[9] Götz J., Chen F., van Dorpe J., Nitsch R.M. (2001). Formation of neurofibrillary tangles in P301l tau transgenic mice induced by Abeta 42 fibrils. Science.

[10] Ferrari A., Hoerndli F., Baechi T., Nitsch R.M., Götz J. (2003). Beta-Amyloid induces paired helical filament-like tau filaments in tissue culture. J. Biol. Chem..

[11] De Felice F.G., Wu D., Lambert M.P., Fernandez S.J., Velasco P.T., Lacor P.N., Bigio E.H., Jerecic J., Acton P.J., Shughrue P.J. (2008). Alzheimer’s disease-type neuronal tau hyperphosphorylation induced by A beta oligomers. Neurobiol. Aging.

[12] Ittner L.M., Ke Y.D., Delerue F., Bi M., Gladbach A., van Eersel J., Wölfing H., Chieng B.C., Christie M.J., Napier I.A. (2010). Dendritic function of tau mediates amyloid-beta toxicity in Alzheimer’s disease mouse models. Cell.

[13] Chabrier M.A., Blurton-Jones M., Agazaryan A.A., Nerhus J.L., Martinez-Coria H., LaFerla F.M. (2012). Soluble aβ promotes wild-type tau pathology in vivo. J. Neurosci..

[14] Pensalfini A., Albay R., Rasool S., Wu J.W., Hatami A., Arai H., Margol L., Milton S., Poon W.W., Corrada M.M. (2014). Intracellular amyloid and the neuronal origin of Alzheimer neuritic plaques. Neurobiol. Dis..

[15] Scarmeas N., Luchsinger J.A., Schupf N., Brickman A.M., Cosentino S., Tang M.X., Stern Y. (2009). Physical activity, diet, and risk of Alzheimer disease. JAMA.

[16] Stranahan A.M., Martin B., Maudsley S. (2012). Anti-inflammatory effects of physical activity in relationship to improved cognitive status in humans and mouse models of Alzheimer’s disease. Curr. Alzheimer Res..

[17] Souza L.C., Filho C.B., Goes A.T.R., Fabbro L.D., de Gomes M.G., Savegnago L., Oliveira M.S., Jesse C.R. (2013). Neuroprotective effect of physical exercise in a mouse model of Alzheimer’s disease induced by β-amyloid_1-40_ peptide. Neurotox. Res..

[18] García-Mesa Y., López-Ramos J.C., Giménez-Llort L., Revilla S., Guerra R., Gruart A., Laferla F.M., Cristòfol R., Delgado-García J.M., Sanfeliu C. (2011). Physical exercise protects against Alzheimer’s disease in 3xTg-AD mice. J. Alzheimers Dis. JAD.

[19] Pena G.S., Paez H.G., Johnson T.K., Halle J.L., Carzoli J.P., Visavadiya N.P., Zourdos M.C., Whitehurst M.A., Khamoui A.V. (2020). Hippocampal Growth Factor and Myokine Cathepsin B Expression following Aerobic and Resistance Training in 3xTg-AD Mice. Int. J. Chronic Dis..

[20] Liu Y., Chu J.M.T., Yan T., Zhang Y., Chen Y., Chang R.C.C., Wong G.T.C. (2020). Short-term resistance exercise inhibits neuroinflammation and attenuates neuropathological changes in 3xTg Alzheimer’s disease mice. J. Neuroinflammation.

[21] Kim D., Cho J., Kang H. (2019). Protective effect of exercise training against the progression of Alzheimer’s disease in 3xTg-AD mice. Behav. Brain Res..

[22] Do K., Laing B.T., Landry T., Bunner W., Mersaud N., Matsubara T., Li P., Yuan Y., Lu Q., Huang H. (2018). The effects of exercise on hypothalamic neurodegeneration of Alzheimer’s disease mouse model. PLoS ONE.

[23] Kim D., Cho J., Lee I., Jin Y., Kang H. (2017). Exercise Attenuates High-Fat Diet-induced Disease Progression in 3xTg-AD Mice. Med. Sci. Sports Exerc..

[24] Li F., Gong Q., Dong H., Shi J. (2012). Resveratrol, a neuroprotective supplement for Alzheimer’s disease. Curr. Pharm. Des..

[25] Kim H.J., Lee K.W., Lee H.J. (2007). Protective effects of piceatannol against beta-amyloid-induced neuronal cell death. Ann. N. Y. Acad. Sci..

[26] Karuppagounder S.S., Pinto J.T., Xu H., Chen H.-L., Beal M.F., Gibson G.E. (2009). Dietary supplementation with resveratrol reduces plaque pathology in a transgenic model of Alzheimer’s disease. Neurochem. Int..

[27] Wang J., Ho L., Zhao W., Ono K., Rosensweig C., Chen L., Humala N., Teplow D.B., Pasinetti G.M. (2008). Grape-derived polyphenolics prevent Abeta oligomerization and attenuate cognitive deterioration in a mouse model of Alzheimer’s disease. J. Neurosci..

[28] Capiralla H., Vingtdeux V., Zhao H., Sankowski R., Al-Abed Y., Davies P., Marambaud P. (2012). Resveratrol mitigates lipopolysaccharide- and Aβ-mediated microglial inflammation by inhibiting the TLR4/NF-κB/STAT signaling cascade. J. Neurochem..

[29] Ladiwala A.R.A., Lin J.C., Bale S.S., Marcelino-Cruz A.M., Bhattacharya M., Dordick J.S., Tessier P.M. (2010). Resveratrol selectively remodels soluble oligomers and fibrils of amyloid Abeta into off-pathway conformers. J. Biol. Chem..

[30] Rahvar M., Nikseresht M., Shafiee S.M., Naghibalhossaini F., Rasti M., Panjehshahin M.R., Owji A.A. (2011). Effect of oral resveratrol on the BDNF gene expression in the hippocampus of the rat brain. Neurochem. Res..

[31] Shojaei S., Panjehshahin M.R., Shafiee S.M., Khoshdel Z., Borji M., Ghasempour G., Owji A.A. (2017). Differential Effects of Resveratrol on the Expression of Brain-Derived Neurotrophic Factor Transcripts and Protein in the Hippocampus of Rat Brain. Iran. J. Med. Sci..

[32] Park H.R., Kong K.H., Yu B.P., Mattson M.P., Lee J. (2012). Resveratrol inhibits the proliferation of neural progenitor cells and hippocampal neurogenesis. J. Biol. Chem..

[33] Cowansage K.K., LeDoux J.E., Monfils M.-H. (2010). Brain-derived neurotrophic factor: A dynamic gatekeeper of neural plasticity. Curr. Mol. Pharmacol..

[34] Esfandiarei M., Hoxha B., Talley N.A., Anderson M.R., Alkhouli M.F., Squire M.A., Eckman D.M., Babu J.R., Lopaschuk G.D., Broderick T.L. (2019). Beneficial effects of resveratrol and exercise training on cardiac and aortic function and structure in the 3xTg mouse model of Alzheimer’s disease. Drug Des. Devel. Ther..

[35] Alkhouli M.F., Hung J., Squire M., Anderson M., Castro M., Babu J.R., Al-Nakkash L., Broderick T.L., Plochocki J.H. (2019). Exercise and resveratrol increase fracture resistance in the 3xTg-AD mouse model of Alzheimer’s disease. BMC Complement. Altern. Med..

[36] Billings L.M., Oddo S., Green K.N., McGaugh J.L., LaFerla F.M. (2005). Intraneuronal Abeta causes the onset of early Alzheimer’s disease-related cognitive deficits in transgenic mice. Neuron.

[37] Oddo S., Caccamo A., Shepherd J.D., Murphy M.P., Golde T.E., Kayed R., Metherate R., Mattson M.P., Akbari Y., LaFerla F.M. (2003). Triple-transgenic model of Alzheimer’s disease with plaques and tangles: Intracellular Abeta and synaptic dysfunction. Neuron.

[38] Zheng C., Geetha T., Gearing M., Babu J.R. (2015). Amyloid β-abrogated TrkA ubiquitination in PC12 cells analogous to Alzheimer’s disease. J. Neurochem..

[39] Crews L., Tsigelny I., Hashimoto M., Masliah E. (2009). Role of synucleins in Alzheimer’s disease. Neurotox. Res..

[40] Salminen A., Kaarniranta K., Haapasalo A., Hiltunen M., Soininen H., Alafuzoff I. (2012). Emerging role of p62/sequestosome-1 in the pathogenesis of Alzheimer’s disease. Prog. Neurobiol..

[41] Su H., Wang X. (2011). Autophagy and p62 in cardiac protein quality control. Autophagy.

[42] Caccamo A., Ferreira E., Branca C., Oddo S. (2017). p62 improves AD-like pathology by increasing autophagy. Mol. Psychiatry.

[43] Sawda C., Moussa C., Turner R.S. (2017). Resveratrol for Alzheimer’s disease. Ann. N. Y. Acad. Sci..

[44] Morris J.K., Vidoni E.D., Johnson D.K., Van Sciver A., Mahnken J.D., Honea R.A., Wilkins H.M., Brooks W.M., Billinger S.A., Swerdlow R.H. (2017). Aerobic exercise for Alzheimer’s disease: A randomized controlled pilot trial. PLoS ONE.

[45] Cass S.P. (2017). Alzheimer’s Disease and Exercise: A Literature Review. Curr. Sports Med. Rep..

[46] Heneka M.T., O’Banion M.K. (2007). Inflammatory processes in Alzheimer’s disease. J. Neuroimmunol..

[47] Velez-Pardo C., Ospina G.G., Jimenez del Rio M. (2002). Abeta[25-35] peptide and iron promote apoptosis in lymphocytes by an oxidative stress mechanism: Involvement of H2O2, caspase-3, NF-kappaB, p53 and c-Jun. Neurotoxicology.

[48] Tansey M.G., McCoy M.K., Frank-Cannon T.C. (2007). Neuroinflammatory mechanisms in Parkinson’s disease: Potential environmental triggers, pathways, and targets for early therapeutic intervention. Exp. Neurol..

[49] Lasagna-Reeves C.A., Kayed R. (2011). Astrocytes contain amyloid-β annular protofibrils in Alzheimer’s disease brains. FEBS Lett..

[50] Saiko P., Szakmary A., Jaeger W., Szekeres T. (2008). Resveratrol and its analogs: Defense against cancer, coronary disease and neurodegenerative maladies or just a fad?. Mutat. Res..

[51] Alexe D.-M., Syridou G., Petridou E.T. (2006). Determinants of early life leptin levels and later life degenerative outcomes. Clin. Med. Res..

[52] Knight E.M., Martins I.V.A., Gümüsgöz S., Allan S.M., Lawrence C.B. (2014). High-fat diet-induced memory impairment in triple-transgenic Alzheimer’s disease (3xTgAD) mice is independent of changes in amyloid and tau pathology. Neurobiol. Aging.

[53] Moraska A., Deak T., Spencer R.L., Roth D., Fleshner M. (2000). Treadmill running produces both positive and negative physiological adaptations in Sprague-Dawley rats. Am. J. Physiol. Regul. Integr. Comp. Physiol..

[54] Lyons C.E., Bartolomucci A. (2020). Stress and Alzheimer’s disease: A senescence link?. Neurosci. Biobehav. Rev..

[55] de la Rubia Ortí J.E., Sancho Castillo S., Benlloch M., Julián Rochina M., Corchón Arreche S., García-Pardo M.P. (2017). Impact of the Relationship of Stress and the Immune System in the Appearance of Alzheimer’s Disease. J. Alzheimers Dis. JAD.

[56] Lasagna-Reeves C.A., Castillo-Carranza D.L., Sengupta U., Sarmiento J., Troncoso J., Jackson G.R., Kayed R. (2012). Identification of oligomers at early stages of tau aggregation in Alzheimer’s disease. FASEB J. Off. Publ. Fed. Am. Soc. Exp. Biol..

[57] Ovsepian S.V., O’Leary V.B., Zaborsky L., Ntziachristos V., Dolly J.O. (2018). Synaptic vesicle cycle and amyloid β: Biting the hand that feeds. Alzheimers Dement..

[58] Ovsepian S.V., O’Leary V.B., Zaborsky L., Ntziachristos V., Dolly J.O. (2019). Amyloid plaques of Alzheimer’s disease as hotspots of glutamatergic activity. Neuroscientist.

[59] Li J., Feng L., Xing Y., Wang Y., Du L., Xu C., Cao J., Wang Q., Fan S., Liu Q. (2014). Radioprotective and antioxidant effect of resveratrol in hippocampus by activating Sirt1. Int. J. Mol. Sci..

[60] Rege S.D., Geetha T., Broderick T.L., Babu J.R. (2015). Resveratrol protects β amyloid-induced oxidative damage and memory associated proteins in H19-7 hippocampal neuronal cells. Curr. Alzheimer Res..

[61] Vingtdeux V., Giliberto L., Zhao H., Chandakkar P., Wu Q., Simon J.E., Janle E.M., Lobo J., Ferruzzi M.G., Davies P. (2010). AMP-activated protein kinase signaling activation by resveratrol modulates amyloid-beta peptide metabolism. J. Biol. Chem..

[62] Pallàs M., Ortuño-Sahagún D., Andrés-Benito P., Ponce-Regalado M.D., Rojas-Mayorquín A.E. (2014). Resveratrol in epilepsy: Preventive or treatment opportunities?. Front. Biosci. Landmark Ed..

[63] Zhao T., Zeng Y., Kermode A.R. (2012). A plant cell-based system that predicts aβ42 misfolding: Potential as a drug discovery tool for Alzheimer’s disease. Mol. Genet. Metab..

[64] Soylemez S., Gurdal H., Sepici A., Akar F. (2008). The effect of long-term resveratrol treatment on relaxation to estrogen in aortae from male and female rats: Role of nitric oxide and superoxide. Vascul. Pharmacol..

[65] Saleh M.C., Connell B.J., Saleh T.M. (2013). Resveratrol induced neuroprotection is mediated via both estrogen receptor subtypes, ER(α) and ER(β). Neurosci. Lett..

[66] Feng Y., Cui Y., Gao J.-L., Li R., Jiang X.-H., Tian Y.-X., Wang K.-J., Li M.-H., Zhang H.-A., Cui J.-Z. (2016). Neuroprotective effects of resveratrol against traumatic brain injury in rats: Involvement of synaptic proteins and neuronal autophagy. Mol. Med. Rep..

[67] Kayed R., Head E., Thompson J.L., McIntire T.M., Milton S.C., Cotman C.W., Glabe C.G. (2003). Common structure of soluble amyloid oligomers implies common mechanism of pathogenesis. Science.

[68] Schweiger S., Matthes F., Posey K., Kickstein E., Weber S., Hettich M.M., Pfurtscheller S., Ehninger D., Schneider R., Krauß S. (2017). Resveratrol induces dephosphorylation of Tau by interfering with the MID1-PP2A complex. Sci. Rep..

[69] Giménez-Llort L., García Y., Buccieri K., Revilla S., Suñol C., Cristofol R., Sanfeliu C. (2010). Gender-Specific Neuroimmunoendocrine Response to Treadmill Exercise in 3xTg-AD Mice. Int. J. Alzheimers Dis..

[70] Ruschak A.M., Miranker A.D. (2009). The role of prefibrillar structures in the assembly of a peptide amyloid. J. Mol. Biol..

[71] Lee J., Culyba E.K., Powers E.T., Kelly J.W. (2011). Amyloid-β forms fibrils by nucleated conformational conversion of oligomers. Nat. Chem. Biol..

[72] Patterson K.R., Remmers C., Fu Y., Brooker S., Kanaan N.M., Vana L., Ward S., Reyes J.F., Philibert K., Glucksman M.J. (2011). Characterization of prefibrillar Tau oligomers in vitro and in Alzheimer disease. J. Biol. Chem..

[73] Maeda S., Sahara N., Saito Y., Murayama S., Ikai A., Takashima A. (2006). Increased levels of granular tau oligomers: An early sign of brain aging and Alzheimer’s disease. Neurosci. Res..

[74] Mattson M.P., Maudsley S., Martin B. (2004). BDNF and 5-HT: A dynamic duo in age-related neuronal plasticity and neurodegenerative disorders. Trends Neurosci..

[75] Phillips H.S., Hains J.M., Armanini M., Laramee G.R., Johnson S.A., Winslow J.W. (1991). BDNF mRNA is decreased in the hippocampus of individuals with Alzheimer’s disease. Neuron.

[76] Holsinger R.M., Schnarr J., Henry P., Castelo V.T., Fahnestock M. (2000). Quantitation of BDNF mRNA in human parietal cortex by competitive reverse transcription-polymerase chain reaction: Decreased levels in Alzheimer’s disease. Brain Res. Mol. Brain Res..

[77] Hock C., Heese K., Müller-Spahn F., Huber P., Riesen W., Nitsch R.M., Otten U. (2000). Increased cerebrospinal fluid levels of neurotrophin 3 (NT-3) in elderly patients with major depression. Mol. Psychiatry.

[78] Michalski B., Fahnestock M. (2003). Pro-brain-derived neurotrophic factor is decreased in parietal cortex in Alzheimer’s disease. Brain Res. Mol. Brain Res..

[79] Yasutake C., Kuroda K., Yanagawa T., Okamura T., Yoneda H. (2006). Serum BDNF, TNF-alpha and IL-1beta levels in dementia patients: Comparison between Alzheimer’s disease and vascular dementia. Eur. Arch. Psychiatry Clin. Neurosci..

[80] Parikh V., Howe W.M., Welchko R.M., Naughton S.X., D’Amore D.E., Han D.H., Deo M., Turner D.L., Sarter M. (2013). Diminished trkA receptor signaling reveals cholinergic-attentional vulnerability of aging. Eur. J. Neurosci..

[81] Hellweg R., Gericke C.A., Jendroska K., Hartung H.D., Cervós-Navarro J. (1998). NGF content in the cerebral cortex of non-demented patients with amyloid-plaques and in symptomatic Alzheimer’s disease. Int. J. Dev. Neurosci..

[82] Gylys K.H., Fein J.A., Yang F., Wiley D.J., Miller C.A., Cole G.M. (2004). Synaptic changes in Alzheimer’s disease: Increased amyloid-beta and gliosis in surviving terminals is accompanied by decreased PSD-95 fluorescence. Am. J. Pathol..

[83] Love S., Siew L.K., Dawbarn D., Wilcock G.K., Ben-Shlomo Y., Allen S.J. (2006). Premorbid effects of APOE on synaptic proteins in human temporal neocortex. Neurobiol. Aging.

[84] Proctor D.T., Coulson E.J., Dodd P.R. (2010). Reduction in post-synaptic scaffolding PSD-95 and SAP-102 protein levels in the Alzheimer inferior temporal cortex is correlated with disease pathology. J. Alzheimers Dis. JAD.

[85] Okabe S. (2007). Molecular anatomy of the postsynaptic density. Mol. Cell. Neurosci..

[86] Sheng M., Kim M.J. (2002). Postsynaptic signaling and plasticity mechanisms. Science.

[87] Selkoe D.J. (2002). Alzheimer’s disease is a synaptic failure. Science.

[88] Arendt T. (2009). Synaptic degeneration in Alzheimer’s disease. Acta Neuropathol. (Berl.).

[89] Neves G., Cooke S.F., Bliss T.V.P. (2008). Synaptic plasticity, memory and the hippocampus: A neural network approach to causality. Nat. Rev. Neurosci..

[90] Griffin É.W., Mullally S., Foley C., Warmington S.A., O’Mara S.M., Kelly A.M. (2011). Aerobic exercise improves hippocampal function and increases BDNF in the serum of young adult males. Physiol. Behav..

[91] Cataldo A.M., Peterhoff C.M., Troncoso J.C., Gomez-Isla T., Hyman B.T., Nixon R.A. (2000). Endocytic pathway abnormalities precede amyloid beta deposition in sporadic Alzheimer’s disease and Down syndrome: Differential effects of APOE genotype and presenilin mutations. Am. J. Pathol..

[92] Pickford F., Masliah E., Britschgi M., Lucin K., Narasimhan R., Jaeger P.A., Small S., Spencer B., Rockenstein E., Levine B. (2008). The autophagy-related protein beclin 1 shows reduced expression in early Alzheimer disease and regulates amyloid β accumulation in mice. J. Clin. Investig..

[93] Funderburk S.F., Marcellino B.K., Yue Z. (2010). Cell “self-eating” (autophagy) mechanism in Alzheimer’s disease. Mt. Sinai J. Med. N. Y..

[94] Jaeger P.A., Wyss-Coray T. (2010). Beclin 1 complex in autophagy and Alzheimer disease. Arch. Neurol..

[95] Morel E., Chamoun Z., Lasiecka Z.M., Chan R.B., Williamson R.L., Vetanovetz C., Dall’Armi C., Simoes S., Point Du Jour K.S., McCabe B.D. (2013). Phosphatidylinositol-3-phosphate regulates sorting and processing of amyloid precursor protein through the endosomal system. Nat. Commun..

[96] Jiang S., Li Y., Zhang X., Bu G., Xu H., Zhang Y. (2014). Trafficking regulation of proteins in Alzheimer’s disease. Mol. Neurodegener..

[97] Nixon R.A. (2007). Autophagy, amyloidogenesis and Alzheimer disease. J. Cell Sci..

[98] Morishima-Kawashima M., Hasegawa M., Takio K., Suzuki M., Titani K., Ihara Y. (1993). Ubiquitin is conjugated with amino-terminally processed tau in paired helical filaments. Neuron.

[99] Ramesh Babu J., Lamar Seibenhener M., Peng J., Strom A.-L., Kemppainen R., Cox N., Zhu H., Wooten M.C., Diaz-Meco M.T., Moscat J. (2008). Genetic inactivation of p62 leads to accumulation of hyperphosphorylated tau and neurodegeneration. J. Neurochem..

[100] Kuusisto E., Salminen A., Alafuzoff I. (2001). Ubiquitin-binding protein p62 is present in neuronal and glial inclusions in human tauopathies and synucleinopathies. Neuroreport.

[101] Du Y., Wooten M.C., Gearing M., Wooten M.W. (2009). Age-associated oxidative damage to the p62 promoter: Implications for Alzheimer disease. Free Radic. Biol. Med..

[102] Høydal M.A., Wisløff U., Kemi O.J., Ellingsen O. (2007). Running speed and maximal oxygen uptake in rats and mice: Practical implications for exercise training. Eur. J. Cardiovasc. Prev. Rehabil..

[103] Dolinsky V.W., Jones K.E., Sidhu R.S., Haykowsky M., Czubryt M.P., Gordon T., Dyck J.R.B. (2012). Improvements in skeletal muscle strength and cardiac function induced by resveratrol during exercise training contribute to enhanced exercise performance in rats. J. Physiol..

[104] Zhang F., Wang H., Wu Q., Lu Y., Nie J., Xie X., Shi J. (2013). Resveratrol protects cortical neurons against microglia-mediated neuroinflammation. Phytother. Res. PTR.

[105] Kothari V., Luo Y., Tornabene T., O’Neill A.M., Greene M.W., Geetha T., Babu J.R. (2017). High fat diet induces brain insulin resistance and cognitive impairment in mice. Biochim. Biophys. Acta Mol. Basis Dis..

